# Environmental sustainability, the forgotten aspect that makes nursing invisible

**DOI:** 10.15649/cuidarte.3480

**Published:** 2024-05-29

**Authors:** Keidis Sulay Ruidiaz Gómez, Ana Sofía Periñan Martínez, María Alejandra Castilla Laurens

**Affiliations:** 1 Universidad del Sinú Elías Bechara Zainúm- Cartagena. Escuela de Enfermería, Cartagena-Colombia. keydis.ruydiaz@unisinu.edu.co Universidad del Sinú Universidad del Sinú Elías Bechara Zainúm- Cartagena Escuela de Enfermería Cartagena Colombia keydis.ruydiaz@unisinu.edu.co; 2 Universidad del Sinú Elías Bechara Zainúm- Cartagena. Escuela de Enfermería, Cartagena-Colombia. anaspm07@hotmail.com Universidad del Sinú Universidad del Sinú Elías Bechara Zainúm- Cartagena Escuela de Enfermería Cartagena Colombia anaspm07@hotmail.com; 3 Universidad del Sinú Elías Bechara Zainúm- Cartagena. Escuela de Enfermería, Cartagena-Colombia. mariale14cas@gmail.com Universidad del Sinú Universidad del Sinú Elías Bechara Zainúm- Cartagena Escuela de Enfermería Cartagena Colombia mariale14cas@gmail.com

Dear editor,

Scientific publications in research journals have adeptly investigated into topics of considerable interest, rendering nursing care more intricate in the face of increasingly thorough investigations into numerous phenomena. This ongoing exploration contributes significantly to the body of knowledge disseminated within the scientific community. Nevertheless, the advent of the global era has presented nursing with novel challenges that persist from the dawn of history parallel to a lingering shadow obscuring the true essence of the profession. An example is environmental nursing, often overlooked from the lens of social sustainability-a neglect that researchers, as minority groups in a relentless struggle, have sidelined. These challenges align with the dynamic demands imposed by society and the mandates of state and/ or governmental parliaments.

Sustainability as progress is one of the goals set out in the Sustainable Development Goals (SDGs) implemented globally in the 2030^1^ agenda, with the aim of working in all necessary areas of the human being and achieving social progress in today's world. SDG 3: health and well-being has been one of the most worked on in the health sector, which gained strength and impact with the appearance of Covid-19, an alarming health situation that left negative health consequences and depressing consequences in the indicators of poverty and inequality; also, SDG 6: clean water and sanitation is prioritized within the health sector, because water is a vital element of the environment that prevents the proliferation of pathogens and prevents infections such as the one presented with the Covid-19 virus2^2^^,^^3^.

Despite global advances, political and state support, there are still 2,000 million people in the world who lack access to water and sanitation and 3,600 million who do not have sanitation services according to the World Bank, which increases the rates of inequality and the spread of diseases. While it may seem utopian, Colombia is one of the countries that ranks ninth in the ranking with 54.78% according to the Centre for Sustainable Development Goals for Latin America and the Caribbean, highlighting that the country has made significant progress in SDGs such as the end of poverty, clean water and sanitation, affordable clean energy and climate action and environment^4^.

Accordingly, in the path of actions built by nursing, the health of individuals and populations remains a priority for the profession. As the International Council of Nurses (ICN) definition of nursing states^5^, "Nursing encompasses health promotion, disease prevention and care of the physically and mentally ill and addresses persons with disabilities of all ages in all health care and other community settings". However, the last principle of protecting the environment seems to be forgotten by nurses today as, in the broad spectrum of care, health promotion, health maintenance and health care responses to individual responses, enhance the deleterious health effects in response to inadequate management of the environment as a means of recovery and wellbeing in individuals.

The aforementioned illuminates nursing from its historical origins as one of the professions promoting environmental sustainability positively impacting the health of human beings. The precursor of nursing, Florence Nightingale, with her environmentalist theory, addresses four concepts 1) health: as a repairer, 2) environment: takes into account external conditions, 3) man: the environment where he develops and his natural defenses , 4) nursing: seeks to cause an environmental impact with the ability to create healthy aspects^6^; these conceptual definitions offered by Nightingale have allowed nurses to participate directly with individuals and groups from the approaches of primary health care, community nursing and the analysis of social determinants with a public health perspective. In addition, it has led the profession to address diverse problems in specific populations, taking into account their cultural characteristics, beliefs, opportunities and threats, in order to improve living conditions in the areas of health, the environment and the economy, through daily work and teamwork on the part of professionals.

Subsequently, the pivotal role of nursing in environmental health is scrutinized through the lenses of healthcare, academia, and research. As environmental management aligns with public health policies and the assessment of environmental risks, nurses are compelled to embrace tools fostering competitiveness, sustainability, and social development. This approach is essential in addressing the environmental impact that significantly influences people's health.

While environmental health or nursing remains somewhat underexplored in the nursing profession, there are global strides in awareness and education. Initiatives like the NurSUS TOOLKIT^7^ project in Europe aim to train change agents, shedding light on environmental degradation impacting the atmospheric layer and declaring a climate emergency, thereby altering disease epidemiological patterns. Additionally, discussion groups like the Alliance of Nurses for Healthy Environments (ANHE) in the United States have been established to equip nurses with the knowledge and skills needed to safeguard environmental health, fostering the creation of resilient and sustainable environments^8^.

The aforesaid observations prompt contemplation on the global endeavors directed towards health intervention, population well-being, and the establishment of support and training programs in environmental policies. It appears that these efforts have garnered significant interest from various disciplines, but nursing, regrettably, still perceives environmental issues as a peripheral concern without immediate relevance in practical applications. Nevertheless, it is crucial to underscore that the environmental realm, grounded in nursing theories, presents a substantial opportunity for the professional advancement of contemporary and future nurses.

In this sense, it is favorable that the nursing profession actively participates in the achievement of the SDG goals, in order to contribute to the integral wellbeing of communities without distinction. At this juncture, it is only appropriate for us to contemplate the unfolding health events, urging the nursing profession to assert itself and assume a pivotal role in environmental sanitation strategies. This involves the delivery of quality medical care, implementation of preventive practices within households and families, and the dissemination of educational initiatives on hygiene habits, among other key aspects. By doing so, the nursing fraternity can actively engage in persistent environmental monitoring, mitigating health disparities that plague communities. Moreover, fostering global partnerships with pivotal organizations such as non-governmental organizations (NGOs), the United Nations (UN), and the World Health Organization (WHO) becomes imperative. Such alliances are instrumental in advancing sustainability in health objectives, ensuring humanitarian resources, upholding human rights, and safeguarding the environment^9^.

Considering what have been said, it becomes evident that environmental nursing, despite its crucial role in healthcare, has been relegated to obscurity. The entities involved in this sphere often confine their activities to a repetitive analysis of the multifaceted environmental issues, lacking effective interventions that address the comprehensive resolution of environmental health demands. In light of this, nursing must pivot its approach, channeling its efforts and healthcare resources towards cultivating environmental awareness among communities, families, and individuals. This shift necessitates a broader, more heterogeneous, and multifactorial perspective to propel nursing into a proactive role in environmental health.

In the current landscape, characterized by diverse perspectives and the countless social phenomena influencing health, there is a pressing need to enhance the professional role of nursing. This enhancement should originate from academic training, focusing on instilling an environmental culture within the curriculum. This involves integrating practical, historical, and legal principles ofthe profession to guide care towards managing environmental resources that impact health or pose risks.

In essence, this concise segment aims to capture the attention of scientific communities, urging them to delve into studies related to environmental nursing, environmental health, and social/climatic changes. These studies contribute to the evolution of nursing care. It is imperative that higher education institutions and multidisciplinary forums for co-creation and debate enrich the collective scientific knowledge. This enrichment will, in turn, benefit diverse societies (social, political, economic, cultural), fostering sustainable work that promotes well-being and relief in vulnerable populations.

Furthermore, in the realm of raising awareness, it is crucial to reshape the perceptions surrounding the nursing profession. The prevalent idealization that confines nursing to clinical/health spaces needs to be challenged. The invisible yet impactful areas of nursing, such as environmental nursing and sustainability for social development, deserve recognition. Without acknowledging nursing's contributions before global leaders, the profession might struggle to gain recognition for its substantial impact on social development and sustainable health.
